# Deliberative Teaching as an Emergent Field: The Challenge of Articulating Diverse Research Agendas to Promote Educational Experiences for Citizenship

**DOI:** 10.3389/fpsyg.2021.660825

**Published:** 2021-06-21

**Authors:** Antonia Larrain, Gabriel Fortes, María Teresa Rojas

**Affiliations:** ^1^Facultad de Psicología, Universidad Alberto Hurtado, Santiago, Chile; ^2^Facultad de Educación, Universidad Alberto Hurtado, Santiago, Chile

**Keywords:** democracy, citizenship, argumentation, deliberative teaching, social inclusion

## Abstract

Democracies are increasingly dependent upon sustainable citizenship, that is, active participation and engagement with the exercising of rights in a field of plural interests, often contradictory and in conflict. This type of citizenship requires not only social inclusion, habits of knowledge, and evidence-based reasoning but also argumentation skills, such as the individual and social capacity to dispute and exercise individual and social rights, and to deal peacefully with sociopolitical conflict. There is empirical evidence that educational deliberative argumentation has a lasting impact on the deep and flexible understanding of knowledge, argumentation skills, and political and citizenship education. However, these three trends of research have developed independently with insufficient synergy. Considering the relevance of deliberative education for contemporaneous democracies and citizenship, in this paper we seek to converge in a field of interlocution, calling it deliberative teaching. Our aim is to propose a way to increase the dialog and collaboration between the diffuse literature on argumentation and education, highlighting both the main theoretical and empirical gaps and challenges that remain and the possibilities to advance our knowledge and the educational impact that this integrating field could offer.

Democracy is not an easy road to take and follow. On the contrary, it is, as far as its realization is concerned in the complex conditions of the contemporary world, a supremely difficult one. (Dewey, 1944/1989, p. 259)

## Introduction

Democracies are being challenged around the globalized world with increasing polarization, institutional crises, and undermined public trust. Although the crisis of democracy has been a topic of discussion for a while now (Merkel, 2014), one could argue that democracies have constantly been in crisis around the world. For instance, violent democracies have been described in the global South for some time, understood as democratic systems in which violence is intimately intertwined with, and functional to, the core of democracy, namely, elections and political participation (Von Holdt, 2014). In particular, democracy, patronage, and violence are complexly entangled to produce “low-intensity citizenships.”

However, it is possible to argue that the recent questioning of the electoral process in the United States, with the consequent risk to trust in electoral institutions, and the incitement to violent riots to interrupt the electoral certification, expresses the crisis at the heart of even a minimal model of democracy (for the electoral regime, see Merkel, 2014) in one of the most developed western democracies. In addition, the inequality in civil rights may also be observed as part of a long-lasting democratic flaw, according to a mid-range notion of democracy (Merkel, 2014). Finally, social and economic inequality, although part of a maximalist notion of democracy, has proven to be a major threat to political sustainability, as demonstrated by social protests and collective and state violence in Chile in 2019.

Democracy needs not only an electoral regime and institutional guarantee of human and civil rights, or “politics” – according to Mouffe (2014) – but also “the political,” that is, civic practices involved in the unfolding of power, particularly practices of dispute and dealing with conflicts. According to an agonistic notion of democracy (see DesRoches and Ruitenberg, 2018, p. 150), strong democracies, in whatever form, need citizens to be engaged in embodying their often conflicting and contradictory interests and struggling to exercise their rights to participate in and dispute decision making from different social identities and voices. Consequently, we understand the difference, tension, disagreement, and contradiction, not as a flaw of democracies but as one of their conditions: “It is only when the ineradicable character of division and antagonism is recognized that it is possible to think in a properly political manner and to face the challenge confronting democratic politics.”

Moreover, democracies are increasingly challenged by specific contemporaneous forms of conflict (global crises) and social communication. New challenges, such as environmental sustainability, global pandemics and economic restrictions, and growing awareness of social, gender, ethnic, economic, and other types of injustice, are accelerated by the development and transformation of communications technology. These challenges demand urgent social action in tensioned and conflicting fields, threatening the sustainability of our ways of life, including not only environmental but also political sustainability. Therefore, the question of sustainability, in general, is central to a contemporaneous notion of democracy (Kyle, 2020), that is, whether present generations are able to deal with these kinds of global crises without risking the living conditions of future generations.

Acknowledging that democracy always involves conflict – and that most of the contemporaneous conflicts we face need to be carefully dealt with to avoid risking the common way of life of future generations – points to a special notion of citizenship. We call this sustainable citizenship (Kurian et al., 2014), understanding it as active participation and engagement with the exercising of rights in a field of plural interests, often contradictory and in conflict, in a way that allows, without dissolving difference, social, cultural, political, economic, and environmental needs to be addressed. It is digital citizenship, insofar as it presupposes active, critical, and responsible engagement with digital technologies that are respectful to human rights. Sustainable citizenship, therefore, is focused on not just participation, or the experience of social conflict as a natural accompaniment to democracy: it is also focused on solving crucial and urgent problems (without dissolving conflict) in a legitimate way. This is particularly important in violent democracies in which social conflict may all too easily become violently elaborated. In these cases, a focus on sustainability in citizenship education is central, or on how to deal effectively and legitimately with pressing problems given the conflicting nature of social life. From this perspective, and especially for violent democracies, neither deliberative citizens, aspiring to solve differences and achieve social rationality, nor agonistic ones, seeking to live emotionally in social difference and plurality, are appropriate. We need something in between: citizens seeking to deal with conflicting positions and emotions in order to address urgent and pressing needs in a sustainable way.

Sustainable citizenship, understood in this way, involves the practice of articulation of a wide range of differences to define and achieve common goals, which require specific individual and social competencies but also institutional design and particular habits. Authors, such as Kurian et al. (2014), have pointed to the role that dialectical deliberation plays in sustainable citizenship, or the deliberation of key controversies as the foundation of citizenship practices, which resonates with a deliberative notion of democracy. Therefore, regardless of whether or not a deliberative democracy is realistic or desirable (see Ryfe, 2005; DesRoches and Ruitenberg, 2018), it is clear that specific individual and social competencies are needed to make our political and environmental worlds sustainable. These competencies include, but are not necessarily limited to: being engaged and willing to participate; being able to argue our points and dispute our interests; being able to understand and evaluate possible arguments; recognizing and conceding others’ good arguments; being strategic in arguments and understanding others’ strategies; articulating with different people; representing and legitimating others’ worldviews; understanding and legitimating different types of knowledge; selecting epistemic authorities to trust in; and positioning ourselves as political and emotional actors among others. Therefore, one could argue that sustainable citizenship requires, both at a collective and individual level, political competencies, argumentation skills, content and epistemological knowledge, in addition to emotional and ethical dispositions.

Empirical evidence, however, produced through different lines of research, points to the experience of deliberative argumentation as a practice that promotes political and civic competencies (Andersson, 2015), argumentation skills (Felton et al., 2015), knowledge (Asterhan and Schwarz, 2016), and social inclusion (Aronson and Bridgeman, 1979). In other words, deliberative argumentation is a transversal key educational practice for contemporaneous citizenship, which has been recognized as such for a while (see Michaels et al., 2008). Why, if this is the case, has deliberative argumentation not been a clear educational goal worldwide? Different answers are available. First, it is a pedagogical practice that requires a sophisticated pedagogical design and expertise (Andriessen and Schwarz, 2009), which has not typically been considered in national curriculums or initial and in-service formation. Second, high-stakes accountability policies undermine the possibilities of schools and teachers innovating regarding argumentative pedagogical designs (Katsh-Singer et al., 2016). Third, research in the area of deliberative argumentation has been dispersed and fragmented, with insufficient synergy, drawing on different theoretical traditions and using different concepts and labels, and with limited capacity to build on one another and influence public opinion.

The aim of this paper is to outline the need to inscribe differently and, thus far, disperse research related to deliberative argumentation in education, under the same field of interlocution. We propose to consider deliberative teaching as a family of pedagogical practices, in order to visualize their contribution to multiple benefits relevant to citizenship. This does not mean that, in doing so, different approaches within this field will be homogenized; on the contrary, the recognition of common ground allows productive dispute and discussion, as well as building up one another’s insights and illuminating knowledge gaps within the field, which, in turn, strengthens the possibilities to impact educational policies and agendas. The proposal does not imply a particular commitment to a deliberative view of democracy, as we have stated. The assumption on which the paper is based is that we need specific educational experiences through which the competencies needed to exercise sustainable citizenship and democratic life can be performed and, in turn, developed.

## Deliberative Argumentation and Sustainable Citizenship: Where is the Evidence?

### Deliberative Argumentation and Argumentation Skills

There is a line of research in developmental psychology that has focused on evaluating, through experimental design, the effect of arguing and thinking with others in educational contexts, especially among peers, on the development of argumentation skills. For instance, Kuhn et al. (1997) asked sixth-graders and young adults to discuss weekly their opinions on capital punishment with different partners in order to reach a consensus. The results showed that, although participants’ opinions were highly stable, there were gains in the range and quality of the grounds of arguments that participants produced, and in their ability to consider two sides of the issue and be aware of the coexistence of multiple views. It is important to note that the aim was not for participants to persuade one another but rather to discuss their views and reach a consensus, corresponding more to a goal of reaching an agreement and understanding than winning the argument. In fact, Felton and Kuhn (2001) report analysis of dialogs showing that younger adults were less focused on undermining partners’ arguments than on clarifying and elaborating upon them.

Kuhn and Crowell (2011) conducted a three-year longitudinal study, also with sixth-graders, in which students had to argue to prepare whole-class debates on social issues, first in face-to-face small groups and then through online dialogs. The results show that students significantly improved their argument quality in post-test written essays, when compared to a comparison teacher-led group. Again, students did not have to argue to win; rather, they had to develop arguments and counter-arguments and evaluate them on several occasions. Only at the end of each topic did they have to argue to win, not through persuasion but through the quality of their argumentative moves. Moreover, Crowell and Kuhn (2014) showed how a three-year intervention with sixth-graders on collaborative peer argumentation not only fostered stronger ways to counter-argue but also bridged the gap between initially low- and high-skilled students.

Kuhn and Udell (2003) reported a study with at-risk eight-graders. Through 16 lessons (12 weeks) of goal-based activities, they had to collaboratively develop reasons into an argument and then discuss the opposing side’s reasons; they also had to deliberate over the best counter-arguments and rebuttals, among others, thereby preparing a debate. The results showed the effect on oral argumentation skills, particularly on the ability to formulate counter-arguments that critically address others’ arguments and rebuttals, and on the quality of their individual arguments. Although the final activity was a persuasion debate, the intense argumentation activity involved a more collaborative argumentation oriented toward evaluating and deliberating the best possible counter-arguments and rebuttals.

Iordanou and Kuhn (2020) examined whether middle-school students in a 12-week intervention benefitted more from arguing in pairs (on the use of gas or solar energy) with opposing peers than they did with same-side peers. They had to construct arguments, counter-arguments, and rebuttals for both positions, and evidence regarding both positions was given. In both conditions, the task involved both face-to-face and electronic dialogs and co-constructive argumentation among same-side pairs. Under the opposing-view conditions, students’ persuasive argumentation was also involved because they were instructed to convince the opposing pair that their position was superior. Finally, all of the students participated in whole-class debates. The results show the effects of opposing-view conditions on the quality of arguments and the number of counter-arguments included in post-test essays.

Reznitskaya et al. (2009) reviewed a series of studies in which students were involved in what was called *collaborative reasoning*. In collaborative reasoning, students deliberate together to answer controversial questions regarding key events in the literature stories, with minimal guidance from teachers. Different from the studies of Kuhn and colleagues, students did not argue in preparation for a persuading debate. The results revealed not only the appropriation of oral argumentation skills during the discussions (Anderson et al., 2001) but also the effect of oral argumentation on post-test written individual essays: students tended to include more satisfactory arguments, counter-arguments, and rebuttals than their peers in the control conditions.

Evidence regarding computer-supported argumentation also shows that computer-assisted collaborative peer argumentation has effects on written argument construction (Nussbaum et al., 2004, 2007; Yiong-Hwee and Churchill, 2007; Bouyias and Demetriadis, 2012; Lin et al., 2012; Noroozi et al., 2013, 2016) and argumentation sequences (Jermann and Dillenbourg, 2003; Stegmann et al., 2007), in addition to quality and knowledge of argumentation (Tsovaltzi et al., 2017).

Overall, the message is consistent and has been supported by multiple qualitative studies: peer argumentation, both orally and electronically mediated, prompts students’ capacity to construct arguments and counter-arguments, both to argue with others and to argue individually in written essays. The question is which kind of peer argumentation would have led to these outcomes? Although the empirical studies mentioned above involve different types of tasks, instructions, and goals, it is clear that the argumentation practices described go beyond simple persuasion, as they involve weighting in peer groups for and against arguments and deciding which are the best before persuading others. It is likely that in the studies of Kuhn and colleagues, the preparation of whole-class debates, that is, the anticipation of persuasion, may play a key role in the quality of peers deliberation (Reznitzkaya and Wilkinson, in press), but the argumentation that unfolded was deliberative rather than fully persuasive. Also, the study by Iordanou and Kuhn (2020) shows that real disagreement and discussion of opposing points of view have an additional effect.

To see whether persuasive (arguing to convince) or deliberative (arguing to reach a consensus) goals had an effect on peer dialogs, Felton et al. (2009) conducted a study with Spanish seventh-graders on sources of energy and climate change. While students in the two experimental conditions (one persuasive and one deliberative) were grouped with disagreeing peers in three sessions (one for each dilemma), students in the control condition worked individually. The results show that students in the deliberative condition outperformed students in both the disputative and control conditions. Further analysis of the dialog (Felton et al., 2015, p. 374) revealed that the deliberative goals produced more elaborative and integrative discussions than the persuasive ones, which were shorter and more closed. The authors define persuasion dialog as “an adversarial exchange in which speakers advance incompatible claims with the goal of convincing others to accept their claim”; and deliberative dialog as “a collaborative exchange in which speakers hold incompatible claims and seek to resolve these differences to arrive at a consensual decision.”

In conclusion, although many unresolved questions remain, the above-mentioned evidence suggests that peer deliberative argumentation on social, literary, and socio-scientific issues prompts school-age students’ argumentation skills. It develops key capacities for sustainable citizenship, namely, dealing with controversial matters committed to the quality of arguments.

### Deliberative Argumentation and Knowledge

Systematic experimental evidence is produced at the crossroads between education and psychology, showing that deliberative argumentation in education promotes knowledge understanding and concept development in different curricular disciplines, including maths, science, and history. For instance, Mercer and colleagues (Mercer and Littleton, 2007), informed by sociolinguistic and sociocultural theories, conducted different quasi-experimental studies in schools and with school-age students in the United Kingdom. The results showed how an *exploratory talk* curriculum prompted students’ knowledge in maths and science. Exploratory talk is a type of discourse described when students have to solve problems cooperatively and to engage critically but constructively with one another’s ideas:

Relevant information is offered for joint consideration. Proposals may be challenged and counter-challenged, but if so reasons are given and alternatives are offered. The agreement is sought as a basis for joint decision-making and action. Knowledge is made publicly accountable and reasoning is visible in the talk (Mercer, 2009, p. 184).

Therefore, we argue that exploratory talk involves the deliberative use of argumentation (Felton et al., 2009), insofar as students have to solve problems collaboratively, and, in order to do so, they have to give, and challenge one another’s, reasoning to reach a consensual solution.

Following a more Piagetian design, Howe and colleagues, also in the United Kingdom, conducted a series of controlled experiments to study the effect of conflict and discussion of different perspectives on conceptual understanding. For instance, they conducted an experimental study to investigate the effects of cognitive conflict, socio-cognitive conflict, and imitation on socio-legal thinking, based on students aged between 9 and 12. The results showed that students in the experimental conditions improved significantly more than students in the control condition from pre- to post-tests. Extended modes of reasoning present in both experimental conditions – agreement with conflicting positions, and disagreements and rejections – were systematically and significantly correlated with post-test gains. Tolmie et al. (1993) conducted an experimental study of primary- and middle-school students, the aim of which was to evaluate the relationship between task design (four conditions), dialog, and conceptual understanding of “floating” and “sinking.” The results revealed that the task design had a significant effect on the amount of discussion among groups, which, in turn, was strongly and statistically associated with pre- to post-test knowledge gains. The more productive condition was the one in which students were asked to agree on a prediction, test their predictions, and reach a consensus regarding the explanation for why the objects floated or sunk. The instruction to reach a consensus was, in fact, shown to be key to prompt discussion about contrary ideas and conceptual gains (Howe et al., 2000). Howe (2009) and Howe and Zachariou (2019) showed that pre- to post-test conceptual progress was related not to the group joint constructions that were appropriated and/or accepted by students but to the discussion of different points of view.

In the United States, Michaels et al. (2008) also showed the effects of what they called accountable talk on student learning during school whole-class discussions. Accountable talk involves participants listening and engaging with one another, and extending and building on one another’s contributions. Students make logical connections and are involved in reasoning, formulating, evaluating, and revising arguments and counter-arguments; they also use evidence that is publicly available. Accountable talk, therefore, as the authors state, is characterized by involving intense deliberative argumentation in classroom discussions.

In the field of argumentation and education, Larrain and colleagues, in Chile (Larrain et al., 2018, 2019, 2020), conducted a series of quasi-experimental studies in schools to evaluate the effect of peer argumentation on science learning in middle-school students. Resonating with the studies of Howe and colleagues, they also conducted the correlational analysis to account for the differential effect of dialog (argumentative moves) on learning. Students in each lesson were typically presented with a conceptual problem and asked to work in small groups to decide consensually which was the best possible solution, formulating arguments and counter-arguments. The results show that repetitive experiences of peer argumentation had an effect on conceptual learning (Larrain et al., 2018) and that frequency of individual formulation of argumentative moves, particularly counter-argumentation, predicted learning (Larrain et al., 2018, 2019, 2020).

Kaya (2013) and Aydeniz and Dogan (2016) conducted two studies with pre-service teachers in Turkey to evaluate the effect of teaching through argumentation (through small groups and classroom discussions) on the conceptual understanding of chemical equilibrium. In both cases, experimental argumentative conditions were compared with the control – teacher-led – conditions. The results showed the significant effects of argumentative conditions on student learning.

In Israel, Christa Asterhan and colleagues produced pivotal laboratory-controlled evidence regarding the relation between deliberative argumentation and learning in undergraduate students. In an experimental study (Asterhan and Schwarz, 2007) on scientific conceptual change, they asked students in two groups to collaboratively solve problems on natural selection. In addition, they asked the experimental group receiving the instruction to reach a common solution through a critical in-depth discussion in which they would try to persuade one another and explain their thinking, seeking to reach the best possible solution by supporting and refuting arguments. Students in the experimental (argumentative) condition surpassed students in the control group on pre- to post-test learning gains.

In order to see whether there is a differential effect of deliberative argumentation over persuasive argumentation on learning, Asterhan and Babichenko (2015) compared a disputative style with a deliberative style of peer argumentation, manipulated *via* confederates. They found that students using the deliberative-style condition outperformed those in the disputative condition on individual learning pre- to post-test gains, showing more openness to share their incomplete understandings with their partner. In a follow-up study with online discussions, Asterhan and Hever (2015) replicated the results, discussing the previous ones by Felton et al. (2009), in which no differences between disputative and deliberative conditions regarding post-test learning were found.

Argumentation has also been conceived of as promoting epistemological knowledge relevant to citizenship; however, overall, the experimental empirical evidence supporting this relationship is scant. It is likely that this is linked to the lack of a clear theory of epistemic cognition development (Sandoval et al., 2016) and the assumption that argumentation requires a certain level of epistemological understanding (see Kuhn et al., 2000), but not the other way around.

Findings regarding content knowledge and argumentation skills have been conducted mostly in isolation. Few studies have explored the potentialities of intervention to foster both outcomes, and their relationship has mostly been unexplored. Although effects on skills, and not on knowledge, have been reported (Wecker and Fischer, 2014), there is experimental evidence that deliberative argumentation prompts knowledge and skills together (see Iordanou et al., 2019). Moreover, recently the intertwinement of knowledge and skills has been studied, with the findings showing the effect of deliberative argumentation on knowledge through skills (Larrain et al., 2020).

To summarize, although there are several knowledge gaps in the field (see Asterhan and Schwarz, 2016), there is experimental and quasi-experimental evidence (again, supported by qualitative studies not revised here), produced in different parts of the world, showing the effect of a particular type of argumentation on the conceptual understanding of social, scientific, and language issues. Although not all of the research groups view the dialog types that they study as argumentation and differences remain, we believe that they converge on the study of the effect of “deliberative argumentation” (Felton et al., 2009, 2015; Asterhan and Schwarz, 2016) with argumentation defined as an engagement in critical thinking, elaboration, and reasoning so that students “can build up a shared understanding of the issue at stake instead of merely convincing or changing their own and each other’s beliefs” (Noroozi et al., 2013, p. 60). This is relevant for sustainable citizenship, which is based on the ability to articulate conflicts and differences, considering different alternatives, using the available evidence and knowledge.

### Deliberative Argumentation and Civic and Political Competencies

Political competencies, understood as multidimensional inclinations, competencies, and behaviors, are key to the notion of sustainable citizenship. They involve aspects, such as political engagement, political understanding (political knowledge about theories and current political events), political skills (related to specific ways of political involvement: organizing people, political strategies, political discussion, or discourse), and political participation and democratic virtues, among others (Beaumont et al., 2006; Persson et al., 2020). The teaching of political competencies, however, has been an increasingly relevant but insufficiently investigated issue (see Beaumont et al., 2006; Andersson, 2015; Bennion and Laughlin, 2018). In the past 15 years, however, there has been growing interest in exploring, comprehending, and evaluating the effect of political discussion as a way to develop these competencies. Beaumont et al. (2006) evaluated, through a pre-post survey design, 21 interventions that involved, among other aspects, engaged political discussions. The results show that these interventions prompted political engagement, knowledge, and skills in undergraduate students. The relationship between political discussion and knowledge was also reported in correlational studies based on surveys in adults, showing that both the frequency and level of elaboration predict knowledge (Eveland and Thomson, 2006) and that this relation is mediated by motivation and information, independent of the level of partners’ information (Eveland, 2004). Moreover, there is correlational evidence based on self-reported measures showing that parent–youth political discussions predict youth political knowledge, especially when parents’ political knowledge is high (McIntosh et al., 2007); adult–youth discussions predict youth civic reasoning (Alvis and Metzger, 2020); and classroom discussions predict political knowledge.

Hess and McAvoy (2014) conducted a longitudinal, mixed-method study of high-school social studies courses (21 schools/35 teachers/1,000 students) that included the discussion and deliberation of political topics. Classroom observations, pre- and post-test surveys, and interviews were conducted. The results showed that classrooms in which students were involved more than 20% of the time in the discussion of controversial political issues, with significant student-to-student talk and high levels of participation, reported significantly more interest in politics as a result of taking the course. They were also more likely to enjoy the political talk and were more comfortable with disagreement. Moreover, the authors concluded that these students were more likely to develop into engaged citizens than students in other classes.

Latimer and Hempson (2012) conducted a quasi-experimental study with undergraduates aimed at evaluating deliberative polling methodology on civic engagement (among other variables) measured with pre- and post-test surveys. The results show an effect of a condition (deliberation) on civic engagement. Less straightforward evidence has also been reported: Andersson (2015) conducted an experimental field design with pre- to post-test measures and two conditions – deliberative teaching and the control as usual condition – in three upper-secondary schools in Sweden. Students were surveyed on democratic virtues (communication competence, political efficacy, and future political participation). The results show the effects of deliberative teaching in vocational programs on some democratic virtues: communication competence and political participation in male vocational programs and political efficacy in female vocational programs. In programs of ensuing academic studies, no effect was found. Persson et al. (2020) conducted a replication study in 59 classrooms (1,200 students) aimed at evaluating the effect of deliberative teaching on self-reported civic competence (political interest, knowledge, democratic values, and political discussions). No effect of the condition was found.

These contradictory results are interesting, because they point to the need not to assume but to empirically test the effect of deliberative teaching on civic competencies. However, two aspects are worth noting. First, contrary to the literature on argumentation skills and knowledge, the effect of deliberative teaching on civic and political competence has not been measured beyond self-report surveys. No measures in actual competencies are reported in these studies. This is important because there is evidence that students in active learning classrooms tend to sub-estimate their learning gains, even when they actually learn more (Deslauriers et al., 2019). Second, in both Andersson (2015) and Persson et al. (2020), the difference between the experimental and the control group was not the absence of deliberation but the type of interactions held: while in the experimental groups, students deliberated in small peer-to-peer groups, in the control condition students deliberated first individually and then in whole-class interactions. It can be hypothesized that deliberation was still too present, even between students, in whole-class spaces. Without the control of deliberative moves during lessons, this cannot be ruled out.

To summarize, there is a wide range of empirical evidence suggesting that deliberative argumentation prompts the development of different competencies and skills relevant to sustainable citizenship. The problem is that the empirical evidence remains disperse and fragmented, because even when the research on argumentation skills and knowledge has been conducted within psychology (which is not the case for political competencies, which has mainly been conducted within political and social sciences education), the dialog between these findings is limited. Thus, we lack an integral conception of what deliberative teaching, as a pedagogical practice, can promote, and the joint evaluation of its different benefits is almost non-existent. In each field, we find important knowledge gaps that deserve more research: the effect of different rhetorical styles on skills and knowledge; the effect on social science knowledge, such as human rights, gender, and environmental issues; dosage of interventions and duration of effects; differences according to age, gender, and ethnicity; and learning processes and transfer effects, among others. However, the main challenge is to raise a unified idea of deliberative teaching that clarifies its main characteristics and points clearly to its different benefits, as part of an integral process of teaching and human learning and development. The hope is that such a view could both foster scientific knowledge on the relationship between deliberative teaching and citizenship and impact the political educational agenda more clearly.

## Deliberative Teaching as a Pedagogical Experience for Sustainable Citizenship

### Theoretical Foundations of Deliberative Teaching

#### Deliberation as a Speech Genre

According to Wiggins (1975), in his *Nicomachean Ethics*, Aristotle outlines two-related concepts relevant to the notion of deliberation, namely, *phronesis* (practical wisdom) and *boulesis* (deliberation). Deliberation is understood as a rational process to uncover the best possible means to the desired end (Abizadeh, 2002). It is involved in practical reasoning, which unfolds when a particular and practical problem – for which there is no general and universal answer, and thus it is open to change – requires the best possible decision, from a moral and practical viewpoint (Price, 2011). Although argumentation did not explicitly emerge as being involved in deliberation, from a contemporary point of view it is difficult to conceive of any process of rational evaluation of different alternatives without the use of argumentative language. So, implicitly, argumentation and deliberation have developed as intimately related concepts. Posterior to these classical ideas on deliberation, this term arises again in the context of European Enlightenment, linked to the relationship between free-thinking, democratic values, and public issues, and political decision-making (see Løvlie, 2007).

Deliberation is also a relevant notion in John Dewey’s thinking. He positions deliberation not only at the center of an idea of democracy and public life but also as a crucial part of thinking. He developed the idea of deliberation in contrast to a utilitarian notion of deliberation. Following an Aristotelian tradition, deliberation, for Dewey (1922), unfolds when there is a dilemma and an uncertain future. This is typically the case with practical and moral issues, which are open to decisions that have no clear and absolute answers. Deliberation, according to Dewey (1922, p. 139), “has its beginning in troubled activity and its conclusion in (the) choice of a course of action which straightens it out.” It is involved in the rational imagination and careful evaluation of different alternative courses of action, based on their consequences. However, we also find deliberation in Dewey’s (1910) writings on scientific thinking and education, when a problem may lead to different technical and theoretical solutions, for which, at some point, there is no clear and definitive answer, and for and against need to be imagined and reasoned. Therefore, once a problem or dilemma has been settled, our reading of Dewey (1910, 1916, 1922) suggests that what defines deliberation is not the practical nature of decisions but: (1) the existence of a problem or dilemma that interrupts and resists habitual ways of thinking; (2) a need to decide on alternative ways of solving the problem or act to restart the flow of thinking; (3) the practice of rational evaluation of the for and against of imagined alternatives and their consequences; and (4) an outcome that is uncertain and indeterminate, so there is no absolute better response beforehand. Moreover, although Dewey does not elaborate upon the notion of argumentation, according to our reading it is inherent to his notion of deliberation (point 3). Finally, it is interesting to note that Dewey (1910) raises a model of thinking based on deliberation; in other words, people deliberate not only interpersonally, to resolve their differences or converge on better solutions, but also with themselves, to deal with personal matters, such as the rational reconstruction of experience.

The idea of deliberation presents in the deliberative teaching approach that has emerged in the field of political science and moral education (Englund, 2016) is informed by Habermas’ theory of communicative action and Dewey’s views on deliberation and education for democracy. Here, deliberative teaching is intimately linked to the idea of deliberative democracy, referring to how schools and classrooms resemble wider social spaces (Englund, 2016). They, however, unlike Dewey, explicitly recognize the role of argumentation, understood as a procedure of social participation and negotiation. Deliberative teaching in this tradition emerges when contrary views are expressed and discussed through argumentation, with the goal and will of reaching a consensus while attending to differences. Tolerance and acceptance of others’ views also characterize this way of teaching, in addition to the possibilities to question traditional views and opportunities for students to communicate with one another with less teacher control. Englund (2016, p. 67) is explicit in viewing deliberative communication as “when conflicts, controversies, confrontations, or different views on any issue arise or are observed and pointed out in the classroom,” so he does not restrict deliberation to decisions about courses of action or practical matters.

#### Deliberation and Argumentation

Walton (1990), who has had a critical influence on educational scholars, envisages argumentation as a social and verbal activity to resolve (or try to resolve) a conflict of interest or difference of opinion. While arguments are claims that serve the purpose of defending a position against opposition or challenges, argumentation is a goal-directed activity, which requires arguers to intentionally and explicitly use persuasive tools to advance (or demerit) a viewpoint (Walton, 1990) in a given interactional situation or dialog type (Walton, 2006). Deliberation is conceived of as a particular dialog type, characterized by the collective goal of deciding upon the best course of action through the rational examination of possible alternatives.

From a pragma-dialectical point of view (van Eemeren and Grootendorst, 2004), argumentation is the communicative activity of increasing (or decreasing) the acceptability of a given position through the use of justified claims in opposition to other justified claims. In this sense, it is both a reasoning procedure and a communicative action aimed at convincing or presenting the merits of a set of propositions. Like Walton’s ideas, the emphasis is on contextual constraints that give argumentation a goal-oriented notion. However, among the van Eemeren’s (2013) ideas, the crucial point resides in understanding how argumentative communication types are ratified socially by language usage in prototypical linguistic communities, such as political, organizational, or academic ways of discussion. Although communicative contexts are a combination of different activity types, they can be clustered in different domains of communication by genre, activity type, and concrete speech events. Similarly, deliberation appears as a specific communication genre, a multi-varied cluster of communication, particular in the domain of political communication (van Eemeren and Garssen, 2010; van Eemeren, 2013). Different from other approaches to deliberation, the emphasis relies on the communicative activity type of political agents toward one another (such as presidential debate) in order to convince a popular audience. In van Eemeren’s (2013) words, his idea differs from that of Walton because it combines deliberation as a discussion procedure with another argumentation genre – the debate – setting the public and political sphere as the scene for deliberation to emerge.

Fairclough’s (2017) approach to deliberation and argumentation comes from discourse analysis and political theory. As they point out, there has not yet been a systematic or comprehensive conceptualization of both argumentation and deliberation across different research fields. Their central point (Fairclough, 2017) is that argumentation (along with other language practices) is considered a macro-speech-act type of discourse, while deliberation is a genre within the frame of this macro-level discourse (Fairclough and Mădroane, 2020). While argumentation coexists with narratives, for example, deliberation coexists with negotiation, adjudication, and others. However, argumentation and deliberation are visibly related, as they deal directly with institutionalized decision-making. Taking a discursive approach, they reclaim the role of rhetoric (Fairclough and Fairclough, 2012) in individual and social choice processes between alternative solutions, be it moral, social, or practical problems. For them, rhetoric plays a significant role in how public decision-making is carried out because we cannot dissociate deliberation from its core aspect of persuasion. Most public debates are held by interested agents who have both collective and personal goals; therefore, they can be seen as a rich space for legitimate and illegitimate rhetorical argumentation (Fairclough and Fairclough, 2012). In a sense, this approach approximates both Walton’s type of deliberation dialog, oriented toward decision making on alternative options, and van Eemeren’s ideas on deliberation as a genre for political debate and audience persuasion.

Although there are differences among these approaches, they converge on conceptualizing deliberation as a particular type of communication activity or speech genre (Bakhtin, 1986), with its own goals, participants, addressees, and compositional styles, in which argumentation, as the activity to formulate arguments and counter-arguments to deal with controversial matters, is used for specific purposes. While argumentation is an abstraction, because it always unfolds through specific genres, deliberation is a family of concrete speech practices. Deliberation, then, involves deliberative argumentation, as a type of argumentation whose goal is to critically and jointly persuade, and be persuaded of, the best possible solution to a given controversial issue. Differences arise when considering the issue at stake, because, regardless of whether or not Walton emphasizes the practical aspects of these issues, Fairclough and Fairclough highlight the institutionalized nature of decision making, while van Eemeren and colleagues ascribe it to political issues.

The ideas of deliberation raised so far overemphasize a rational view of the process. We, however, conceptualize argumentation from a dialogical theory of language (Vološinov, 1929/1986), which acknowledges the affective, positioned and evaluative nature of its unfolding. Moreover, as contradiction is explicitly presented, elaborated, and organized through argumentative language, including deliberative argumentation, it involves identity and motivational processes, and political emotions (Ruitenberg, 2009; Bendixen, 2010), which are raised but also organized, shaped, and transformed through discussions.

Based on these points of view, the presence of deliberation in education is not straightforward. However, as we will argue in the next section, we believe that classroom deliberation is a speech genre that should be conceptualized and taken as a guiding principle to design educative experiences to promote sustainable citizenship.

Therefore, from now on, taking the idea of deliberation raised in the previous section, we understand it as a family of speech genres in which speakers carefully, critically, and affectively consider alternative solutions to controversial and dilemmatic open issues (whether practical or not). They do it through the persuasive and affective imagination, formulation, evaluation, and revision of arguments and counter-arguments, and with the aim of reaching a shared (although plural) view on the matter. Again, it is worth noting that people are involved in argumentative practices from their emotional dispositions, identities, and particular experiences. This may be viewed as a potential threat to deliberation, insofar as speakers may be biased by their individual emotional (Kunda, 1990) and cognitive (Mercier and Sperber, 2017) dispositions and virtues (De Brasi, 2020). However, we consider speakers’ affective positionings to be key to engaging and participating in experiences of deliberation that, in turn, are developed by them. Therefore, some authors have emphasized the importance of carefully designing deliberative experiences (Battaly, 2016) to promote individual and collective dispositions.

### Deliberative Teaching as a Field of Experience

We propose to conceive of deliberative teaching as a certain type of educational experience, whether it unfolds in science, language, the arts, maths, social sciences, or civic education, among others. Although deliberative teaching has already been used in the field of political science (also deliberative pedagogy – Shaffer et al., 2017; or deliberative communication – Englund, 2016), with the notion of deliberative democracy as the orienting principle, we propose to borrow the term to articulate a broader and more diverse field of research and professional development in education. As such, deliberative teaching goes beyond a mere intersection of general terms, such as argumentation and education, to summon up initiatives through the common experiences that they offer to students and teachers. The gathering of different initiatives in this field would allow researchers to overcome the fragmentation of our knowledge on the benefits of, and conditions for, this kind of pedagogical experience, enabling a clearer visualization of its potential to promote the development of integral, sustainable, and strong citizens, and thereby – hopefully – achieving a relevant place in educational agendas.

The remarks on Dewey in the previous section are pivotal to our purposes because were deliberation simply a matter of deciding practical problems, some of the revised literature would fall out of the field. Many of the studies revised in the previous sections indeed involve students discussing practical matters and imaginary courses of action, whether moral, such as capital punishment (Kuhn et al., 1997) or characters’ motivations (Reznitskaya et al., 2009), socio-scientific issues, such as energy use (Felton et al., 2015), or political issues, such as democracy, human rights, or gender (Andersson, 2015). However, there are other problem-based studies involving the discussion of decisions based on the evaluation of arguments and counter-arguments, which are not practical but theoretical (related to concepts and explanations rather than what to do – Asterhan and Schwarz, 2007; Howe, 2009; Larrain et al., 2019; among many others). Our positioning here is that these studies also involved deliberation because, from the students’ point of view, they had to decide on dilemmas that were uncertain (they did not have the canonical solutions), and for which they needed to imagine and evaluate critically possible solutions. Moreover, in our view, deliberative teaching has a societal and political value, independent of what is at stake and one’s notion of democracy (i.e., deliberative, agonistic, feminist, or other): deliberation is an experience and opportunity to develop key skills, affective positionings, and knowledge into an active citizenship, which, even in violent democracies, may play a key role.

Deliberative teaching as an experience (or family of experiences) is typically characterized by engaging students in activities in which curricular or extracurricular pedagogical goals are attained through intense peer deliberative argumentation around carefully designed controversial problems. It supposes and promotes an inclusive ethos of respectful collaboration and critical engagement. We follow Dewey (1934, p. 42) and his notion of experience as a meaningful unit of a given stream of life, which is felt as a whole, having its own aesthetic quality:

(…) we have an experience when the material experienced runs its course to fulfillment. Then and only then it is integrated within and demarcated in the general stream of experience from other experiences. A piece of work is finished in a way that is satisfactory; a problem receives its solution; a game is played through; and a situation, whether that of eating a meal, playing a game of chess, carrying on a conversation, writing a book, or taking part in a political campaign, is so rounded out that its close is a consummation and not a cessation. Such an experience is a whole and carries with its own individualizing quality and self-sufficiency. It is an experience.

Therefore, we say that deliberative teaching is an experience, in the sense that, from students’ subjective viewpoints, it is rounded out with aesthetic quality, with an emotional unity organized by the specific situation in which they participate. It involves clear motives, goals, tasks, and endings; it offers opportunities to feel in a certain way and actively engage with meaningfully presented knowledge; and spaces for social dispute and differentiation, social recognition, and mutual appreciation. Deliberation on any matter or issue involves value judgments and, with them, personal biographies and worldviews: “Deliberation is dramatic and active” (Dewey, 1932, p. 275). Therefore, deliberative teaching involves integral subjects in interdependent subjectivating of meaningfully driven activities, through which skills and knowledge are developed as habits. It is, again, not a rational interplay; rather, it opens the opportunity to enact collective and political emotional positionings (Ruitenberg, 2009) that are key to democratic life.

Deliberative teaching as experience is likely to involve many different and complementary learning and developmental processes discussed in the literature so far: socialization and habituation (McIntosh et al., 2007), appropriation (Anderson et al., 2001), internalization (Larrain et al., 2020), reconstruction of cognitive structures (Howe, 2009), deep elaboration (Eveland and Thomson, 2006), and dramatization and role-taking (Ruitenberg, 2009), among others. In this sense, it is also a learning process that integrates different processes of learning and development, bringing different theoretical and epistemological traditions together.

As such, deliberative teaching calls for whole persons to develop integrally different aspects and dimensions of their personalities and subjectivities in the same stream of life. If we fail to see this as a field, as a family of classroom practices providing students with singular experiences and pointing in the same direction, we are left with bits and pieces but we lose the full picture. In so doing, education loses the crucial potential to develop integral, sustainable citizens. Our argument is that teaching sustainable citizenship requires an integrated view – pedagogically implemented – of students’ subjective and academic development, which is precisely what deliberative teaching, given all its reported benefits, can offer.

Therefore, our argument is that by inscribing our research in the field of deliberative teaching, on the one hand, we can more clearly recognize dialoguing research on the conditions, characteristics, and effects of this particular type of pedagogical experience, moving the field consistently forward. On the other hand, the idea of deliberative teaching might help to overcome the abstractness of the idea of argumentation, which, from the perspective of teachers and stakeholders, might be counter-intuitive, while avoiding the fragmentation and confusion that different available but equivalent labels could reproduce.

### Deliberative Teaching, Social Inclusion, and Educational Justice

Deliberative teaching for sustainable citizenship has a twofold relationship with social inclusion in schools. It assumes that schools are spaces of encountering and sociocultural recognition and appreciation of differences (social, sexual, gender, ethnic, body, and among others), where the idea of normality is disputed and symmetrical participation in educational spaces is seen as key to inclusive education (Slee, 2001). At the same time, it could be seen as an experience to promote social justice.

Social inclusion in education is a complex field. Different traditions have put forward different arguments, emphasizing different identitary and subjective aspects of human development as a focus of inclusion. Therefore, when speaking of social inclusion in schools, we are typically pointing out one of many aspects: the inclusion of students with disabilities (Ainscow and Miles, 2008); of socio-economically diverse students (Van Zanten, 2003; Duru-Bellat, 2004; Bonal and Bellei, 2018); the inclusion of gender and sexual diversities (Tinklin et al., 2003), with the concomitant disarticulation of heteronormative cultures within schools (Miller, 2016); and/or the inclusion of ethnically diverse students through the dialogical participation of different national and ethnic group cultures in schools (Dietz, 2012). All of these traditions share a widely acknowledged view of social inclusion as an ethical imperative regarding the role of educational justice in educational systems, offering equal opportunities of participation and recognition and the appreciation of diverse and plural identities of students and their communities, to construct respectful and democratic social relationships and values (Kumashiro, 2001).

The problem is that inclusion is a challenging educational goal, and there is still a gap between these ideal and educational realities (Ainscow et al., 2006). Socioeconomic inclusion is almost impossible in segregated educational systems in which students are separated according to ethnic or socioeconomic conditions (Bonal and Bellei, 2018). There are many studies showing that despite the advances in educational policies to avoid arbitrary gender and sex discrimination in schools, gender gaps, sexism, gender stereotypes and prejudices (Bragg et al., 2018), social exclusion of diverse gender, and sexual identities, still persist (Cumming-Potvin and Martino, 2018). Finally, empirical evidence shows that, although migrant students tend to access education in many countries, they have to face racist and xenophobic practices within schools, in addition to national monocultural curriculums (Sleeter, 2018). In fact, empirical evidence has shown that social or ethnic mixing in schools is not sufficient to deactivate prejudices, stereotypes, and discriminatory practices. On the contrary, different segregation mechanisms may operate (Reay, 2004), such as curricular tracking (Sevilla and Polesel, 2020), groupings by friendship or family influence in school schedules (Reay and Ball, 1998; Van Zanten, 2003).

Fragmentation of the field of social inclusion in education does not help it to advance integrally in educational justice. The different traditions mentioned claim different identities, expressing particular sociopolitical debates. This has had consequences for educational policies, insofar as they have tended to regulate specific aspects of subjective development separately: disability, socioeconomic disadvantage, gender, or ethnic diversity. These policies penetrate schools in a disperse and disarticulate way, reinforcing the stereotypes of teachers and principals, with less impact on schools’ capacity to promote democratic relations in diverse settings (Slee, 2001). Therefore, pedagogical practices offering integral experiences of inclusion in schools are both scarce and compulsory (Frankenberg and Orfield, 2012; Blokland and Nast, 2014).

Social inclusion is not an end in itself (Slee, 2001) but a baseline scenario for basic conditions for educational justice. Following Dewey’s (1916) legacy on democratic education, education involves a process of cultural reconstruction in which all, without exclusion, should find recognition of their different individual experiences and plural identities and their contribution to collective life. The ability to raise common goals, collaboratively and symmetrically, in the absence of dominant hegemonies is what characterizes real inclusive school cultures (Dewey, 1916; Slee, 2001). However, this recognition needs to be institutionally facilitated; it cannot simply be demanded as an ethical mandate relying on students’ individual socio-emotional skills. Beyond putting students together, and following Juvonen et al. (2019), Nishina et al. (2019), and the available empirical evidence (Aronson and Bridgeman, 1979; Sharan, 1980; Graham, 2018; García-Carrión et al., 2020), we argue that schools need to provide students with opportunities to have engaging and meaningful educational experiences of encountering and collaborative thoughtful activity with their peers. This allows them to reconstruct a common frame for identitary articulation (Rojas et al., 2016), thereby promoting friendship (Graham, 2018; Juvonen et al., 2019) and building integrally inclusive schools.

We argue that deliberative teaching, although requiring diversity as a basic condition, can also be conceived of as an inclusive pedagogical experience, that is, enhancing and deepening social inclusion in schools. Deliberative teaching offers possibilities of real encounters and mutual knowledge and recognition, in which real differences are expressed and articulated. On the other hand, it offers spaces to focus on the ideas and arguments, juxtaposing in a meaningful way the worldviews and subjectivities of diverse others, offering the chance to represent others’ perspectives and appreciate their contribution, and in the process breaking down prejudices. This is one way in which peer effects (Van Ewijk and Sleegers, 2010) can act to diminish academic segregation under socially heterogeneous conditions, promoting positive outcomes to both socioculturally disadvantaged (Van Zanten, 2003; Duru-Bellat, 2004; Bonal and Bellei, 2018) and advantaged students (Orfield, 2001; Orfield and Frankenberg, 2013). However, these practices and experiences require a broader view on education for democracy, which can involve an idea of deliberative education. In such a view, coherent with Dewey’s (1916) ideas, deliberation should be seen as a practice beyond classrooms, involving practices of teachers’ professional development, curricular and pedagogical decision making, and practices of articulation of all school actors. More importantly, deliberative teaching practices need to be developed in a broader framework of democratic and educational justice, where everybody’s dignity and experience are valued and used to raise collective norms and values (Feu et al., 2017; Belavi and Murillo, 2020).

## Discussion

The main argument of this paper is that deliberative teaching, as a field grouping diverse research on classroom experiences involving peer deliberative argumentation, can visualize the affordances of these transversal pedagogical practices to promote the development of integral subjects into sustainable citizens. We observe that the different traditions we have explored through the paper are sending a clear but insufficiently heard message: Classroom deliberative argumentation can have crucial benefits for citizenship, whether the object of deliberation is scientific, mathematical, social, artistic, or moral. These benefits become really meaningful for citizenship development only when one overcomes the fragmentation of the different dimensions studied as benefits (argumentation skills, knowledge, politics, and civic competence) and of the different political agendas behind the study of social inclusion in education. The configuration of deliberative teaching as a field can be the first step into a necessary integral view of citizens’ development.

The idea of deliberative teaching is not new. The label has already been used in political science and social science education (Andersson, 2015) and has its roots in Dewey’s thinking. Moreover, research on classroom dialog and deliberative democracy has already been proposed [Erduran and Kaya, 2016; Michaels et al. (2008)]. Our proposal, however, is to use this notion to give to an implicit and disarticulated field a common identity that enhances the possibilities of diverse research traditions and contributions in order to achieve mutual recognition, convergence, and educational impact. Moreover, although we know about the benefits of deliberative teaching, there are still relevant knowledge gaps within each sub-field. For instance, experimental evidence on skills has been found mainly in countries in the global North, and questions about dosage – how long and intense interventions should be – and transfer remain (see Reznitskaya et al., 2012). Evidence on knowledge of social issues, such as human rights and gender have been less well attended, and learning mechanisms are still insufficiently understood (Larrain et al., 2019). The relationship between deliberative argumentation and epistemological knowledge is also a persistent challenge. The effect of deliberation on political competence beyond self-reports, and the differential role of peer interaction (Persson et al., 2020), still need to be appropriately studied. In addition, although the relation between deliberative teaching and social justice makes perfect sense, it has not been extensively empirically studied, mainly because social inclusion research agendas have not yet crossed over to the other research traditions presented here. More importantly, the assumption that deliberative teaching can prompt many benefits at the same time (Iordanou et al., 2019; Larrain et al., 2020), and that these benefits are relevant to sustainable citizenship, still needs to be empirically tested. However, this requires an integrated view on the matter.

It is worth noting that promoting knowledge plays a key role in the deliberative teaching potential to develop sustainable citizens. This is the case not only because of what has already been discussed, for instance, the role of knowledge and scientific literacy in public evidence-based decision making. In addition, we argue that climate action, social inclusion, and justice also require knowledge construction on key issues. For instance, regarding sexual and gender inclusion, if students are told from a moral point of view that they should respect others and avoid discrimination of women or other gender identities, they can accept or reject it, because it has been relayed as a dogma. Instead, if that general idea is transformed into a situated problem and is open to deliberation, with the expression and argumentation of many points of view – even ones that seem unacceptable – this can contribute to a deep understanding of the tensions involved, legitimating normative and legal decisions. This of course imposes tensions on teachers, who may perceive it as difficult to orchestrate and consolidate discussions in which different points of view, including unacceptable ones, are expressed and promoted. Although there have been several initiatives to promote and study professional development for scientific argumentation in classrooms (Osborne et al., 2019), more studies are needed.

Deliberative teaching as pedagogical experiences could be accused of idealism and liberalism, and for good reason. Authors have warned against deliberative pedagogies as disciplining practices for a liberal view of citizens (DesRoches and Ruitenberg, 2018). Michaels et al. (2008) highlight the real experiences of deliberation in classrooms in which social and cultural capital (see Dubet, 2004; Bourdieu, 2011) could differentially shape students’ participation and, in turn, reproduce pre-existing inequalities. Moreover, status and power asymmetries operating in peers’ social relations render deliberation experiences dependent on social structures. This has been illustrated by middle-class students doing better in socially mixed public schools, enlarging the initial differences (Jansson et al., 2020; Mendoza, 2020). We need to pay special attention to prevent deliberative teaching practices from promoting more segregation by institutionalizing and privileging one type of social participation over the diversity of political agency.

However, we think that there are ways to make deliberation central to the educational experience without simply reproducing a certain type of citizen or social inequalities. In other words, deliberative teaching is not necessarily liberal, rational, and elitist. If deliberative discussions are not only cosmetic but also involve as objects of a dispute the social conditions of life and education, we believe that they are still one of the best ways to break students’ asymmetric power positions and agencies. A critical school curriculum, flexible and permeable to diverse life experiences, and a culture of critical and collaborative school governance, prioritizing the redistribution of students’ learning opportunities (Belavi and Murillo, 2020), have been considered key to deliberative professional cultures that increase the probabilities of social inclusion at school (Mabovula, 2009). Therefore, again, deliberative teaching needs to be part of a general framework of education for social justice and democracy at the school level, in which social differences are visible and gaps are acknowledged and not simply accepted.

Deliberative teaching might appear to be unreachable within the current educational systems, organized by high-stakes accountability policies and testing agendas, which in some countries co-exist with mercantile policies. These educational systems have increased their social segregation (Murillo and Martinez-Garrido, 2020), threatening social inclusion goals (Frankenberg and Orfield, 2012; Bonal and Bellei, 2018; Murillo and Martinez-Garrido, 2020), and reducing innovative teaching practices (Mathison and Freeman, 2003; Parcerisa and Falabella, 2017) and curriculum richness (Au, 2007). Deliberative teaching should consider these sociopolitical conditions, identifying how they tension its unfolding (Katsh-Singer et al., 2016; Ydesen et al., 2020) and developing an understanding of how to design situated deliberative teaching practices that consider teachers’ labor conditions (Ball and Olmedo, 2013). Otherwise, it could contribute to concerns among teachers and principals about how to articulate different educational goals (Ryan, 2006, 2010). Furthermore, to imagine deliberative teaching, extracurricular online instances whereby students can interact with students from different schools and backgrounds could be a way to promote social justice within segregated educational systems.

Deliberative teaching, as we understand it, does not imply a notion of deliberative democracy, in which conflicts are rationally solved and the best possible options are achieved. We value and acknowledge conflict and tension as part of any democratic life, with no need to dissolve them. However, we are not arguing for a fully agonistic view of democracy (Mouffe, 2014). As South American scholars, we are committed to the need to recognize conflicts as part of social life, but acknowledging the challenge and the need to handle them in a way that enables a common and sustainable life. Thus, developing skills and political affections to achieve this is key. In that sense, our view of sustainable citizenship departs from a notion of adaptive or individualized citizenship, establishing intimate relations with Veugelers’ (2020) idea of critical–democratic citizenship, or Rapanta et al.’s (2020) notion of culturally literate citizens, both of which recognize argumentative deliberation as a relevant means of education.

The argument that is central to this paper is the need to concur with a field based on the notion of deliberative teaching. Reasonable counter-arguments could be put forward to challenge the proposal, questioning the real effect of a new label, considering that there are ones already available that have failed to solve the fragmentation of current research (for instance, dialogical teaching or inclusive education). Our answer would be that these fields include broader conversations, which are not focused on deliberative argumentation. As we argue that deliberative argumentation has several benefits to citizenship education, this is what we are proposing to bring to the fore as a common ground. So, it is not a question of simply labeling or establishing a new small *feudo* for a given research agenda. It is about inviting more people to take part in the conversation that is already happening but clouded by many other conversations going on in these fields. The invitation is to recognize a non-exclusive and superposing field of interlocution that can provide us with specific affordances to discuss issues of deliberative teaching and its impact on citizenship education.

## Author Contributions

AL, GF, and MR drafted the first version of the manuscript and reviewed the final one. All authors contributed to the article and approved the submitted version.

### Conflict of Interest

The authors declare that the research was conducted in the absence of any commercial or financial relationships that could be construed as a potential conflict of interest.
